# A complex breastfeeding promotion and support intervention in a developing country: study protocol for a randomized clinical trial

**DOI:** 10.1186/1471-2458-14-36

**Published:** 2014-01-15

**Authors:** Mona Nabulsi, Haya Hamadeh, Hani Tamim, Tamar Kabakian, Lama Charafeddine, Nadine Yehya, Durriyah Sinno, Saadieh Sidani

**Affiliations:** 1 Department of Pediatrics and Adolescent Medicine, American University of Beirut, Beirut, Lebanon; 2 Biostatistics Unit, Clinical Research Institute, Faculty of Medicine, American University of Beirut, Beirut, Lebanon; 3 Faculty of Health Sciences, American University of Beirut, Beirut, Lebanon; 4 Olayan School of Business, American University of Beirut, Beirut, Lebanon

**Keywords:** Breastfeeding, Lay support, Cost analysis, Lactation support, Knowledge, Attitudes, Social network, Social support theory

## Abstract

**Background:**

Breastfeeding has countless benefits to mothers, children and community at large, especially in developing countries. Studies from Lebanon report disappointingly low breastfeeding exclusivity and continuation rates. Evidence reveals that antenatal breastfeeding education, professional lactation support, and peer lay support are individually effective at increasing breastfeeding duration and exclusivity, particularly in low-income settings. Given the complex nature of the breastfeeding ecosystem and its barriers in Lebanon, we hypothesize that a complex breastfeeding support intervention, which is centered on the three components mentioned above, would significantly increase breastfeeding rates.

**Methods/Design:**

A multi-center randomized controlled trial. *Study population*: 443 healthy pregnant women in their first trimester will be randomized to control or intervention group. *Intervention*: A “prenatal/postnatal” professional and peer breastfeeding support package continuing till 6 months postpartum, guided by the Social Network and Social Support Theory. Control group will receive standard prenatal and postnatal care. Mothers will be followed up from early pregnancy till five years after delivery. *Outcome measures*: Total and exclusive breastfeeding rates, quality of life at 1, 3 and 6 months postpartum, maternal breastfeeding knowledge and attitudes at 6 months postpartum, maternal exclusive breastfeeding rates of future infants up to five years from baseline, cost-benefit and cost-effectiveness analyses of the intervention. *Statistical analysis*: Descriptive and regression analysis will be conducted under the intention to treat basis using the most recent version of SPSS.

**Discussion:**

Exclusive breastfeeding is a cost-effective public health measure that has a significant impact on infant morbidity and mortality. In a country with limited healthcare resources like Lebanon, developing an effective breastfeeding promotion and support intervention that is easily replicated across various settings becomes a priority. If positive, the results of this study would provide a generalizable model to bolster breastfeeding promotion efforts and contribute to improved child health in Lebanon and the Middle East and North Africa (MENA) region.

**Trial registration:**

Current Controlled Trials ISRCTN17875591

## Background

Despite its countless benefits to children, mothers and community at large, breastfeeding rates in Lebanon continue to be disappointingly low. Previous studies reported acceptable initiation rates varying between 63.8% and 96% [1,2]. However, exclusive breastfeeding is reported in 58.3% of babies less than one month, and in 4.1% to 10.1% of 6-month old infants [3-6]. Only 27.1% of one-year old infants continue to breastfeed [3].

Breastfeeding is associated with reduced infant risk of infections, atopic dermatitis, asthma, obesity, diabetes types 1 and 2, childhood leukemia, sudden infant death syndrome, necrotizing enterocolitis; and with higher Intelligence Quotient and academic performance at 6.5 years of age [7-9]. Moreover, it is associated with decreased maternal risks of diabetes type 2, breast and ovarian cancers, and postpartum depression [7]. As such, breastfeeding is a cost-effective public health measure that has a significant impact on maternal health and infant morbidity and mortality in developing countries [10,11]. Exclusive breastfeeding for 6 months and continued until 11 months of age is the single most effective strategy to improve child survival in developing countries, preventing 13% of under-five mortality [12].

Several cross-sectional studies from Lebanon reported different predictors of low breastfeeding rates. These included lower socio-economic status, Caesarean birth, urban residence, early hospital discharge, mother’s religion, male paediatrician, and hospital practices that hinder breastfeeding like lack of rooming-in of mother and baby, implementing fixed newborn feeding schedules, and offering glucose and water or artificial formula as first feeds instead of breast milk [2,4,13]. A recent qualitative study that explored breastfeeding perceptions and experiences of 36 new mothers who were followed up longitudinally for one year reported several barriers to breastfeeding exclusivity and continuation in the Lebanese context [14]. These included several maternal and community misconceptions such as insufficiency of breast milk and lack of satiety in the baby, breastfeeding causing maternal weight gain or breast sagging, maternal milk being harmful in certain situations such as grief, maternal illness or pregnancy. Moreover, mothers complained of breastfeeding being painful, resulting in sleep deprivation and exhaustion. On the other hand, women who continued breastfeeding for one year were cognizant of the difficulties of breastfeeding and despite this showed determination to succeed and overcome any barrier, relying mostly on family support and proper time management. That study uncovered the need for several interventions that can address the different barriers in the Lebanese context, and the need to empower mothers to overcome them, hoping to improve the existing national breastfeeding exclusivity and continuation rates.

Of the different interventions reported in the literature to improve breastfeeding rates, breastfeeding support, whether professional or lay, was quite effective in increasing breastfeeding duration [15-18]. In particular, lay support with or without a professional component increased the rate of any short- or long-term breastfeeding, as well as the rate of short-term exclusive breastfeeding duration [16]. Lay breastfeeding support has additional health benefits in several aspects of families’ lives such as playing a role in reducing obesity and postpartum depression [17]. Evidence suggests it tends to be more effective in low- and middle-income settings [18]. Similarly, peer support interventions had a significantly greater effect on any breastfeeding in low- or middle-income countries, reducing the risk of not breastfeeding at all by 30%, compared with a reduction of 7% in high-income countries. Moreover, the risk of non-exclusive breastfeeding decreased significantly more in low- or middle-income countries than in high-income countries with these interventions [18]. A recent review of four randomized controlled trials, including one from Syria showed that community-based interventions were significantly associated with an increase in exclusive breastfeeding rates at four and six months after [19]. Interestingly, a mother-to-mother breastfeeding line that was established in Toronto and Nova Scotia to promote and support breastfeeding women succeeded in achieving one hundred percent breastfeeding rate by the end of the third month among the women who participated in The Yarmouth Friendly Feeding Line (YFFL) pilot [20]. Also, a recent Cochrane review that assessed the effectiveness of support for breastfeeding mothers of healthy term babies found that all forms of extra support analyzed together increased the duration of exclusive breastfeeding at 6 months (RR = 0.86; 95% CI: 0.82 to 0.91). Support was more effective in settings with high initiation rates (like Lebanon); with strategies that rely on face-to-face support being more likely to succeed as opposed to support that is offered reactively upon mothers’ request [21].

Breastfeeding education is another intervention that was associated with a significant increase in initiation rates, specifically in low-income USA women as compared to standard of care (RR 1.57, 95% CI: 1.15 to 2.15) [22]. In particular, one-to-one, needs-based, informal repeat sessions, and generic formal antenatal education were effective in increasing breastfeeding rates. Needs-based, informal *peer* support, whether ante-natal or post-natal were particularly effective in increasing initiation rates (RR 4.02, 95% CI: 2.63 to 6.14) [22].

The totality of evidence underscores the heterogeneous and complex nature of breastfeeding barriers in any given setting. Evidence thus suggests that in order to effectively raise breastfeeding rates, there is need for multi-dimensional interventions that simultaneously tackle different aspects of breastfeeding. In this proposed randomized clinical trial, we plan to investigate whether a complex breastfeeding promotion and support intervention starting from early pregnancy is effective in improving six-month breastfeeding exclusivity rates. We hypothesize that a complex intervention composed of several simple interventions that were previously shown to individually improve breastfeeding rates in low- and middle-income countries is more effective than the standard of care in improving breastfeeding exclusivity rate in Lebanon, one of the lowest in the region [23].

Our proposed complex intervention is based on the Social Network and Social Support theory framework, which offers a framework describing pathways through which social ties can influence health [24]. Social networks represent the web of social ties or relationships through which an individual receives social support. Social networks are characterized by the dyadic characteristics of 1) reciprocity, the extent to which support is given and received in a relationship, 2) intensity, the emotional closeness experienced in a relationship, and, 3) complexity, the variety of functions that the relationship serves. In addition, the characteristics of homogeneity in terms of demographic characteristics, geographical dispersion in terms of proximity to the focal person and density in terms of the extent of interaction of members, are used to describe the network as a whole. Social networks represent relationships between people that provide social support as one of their functions. Social support is categorized in four supportive behaviors:

1. Emotional support: conveying that a person is valued and cared for in health promoting ways.

2. Instrumental support: provision of aid and services.

3. Appraisal: provision of information for self-evaluation; constructive feedback.

4. Sharing points of view: offering opinions about how one would handle a situation.

5. Informational support: provision of advice or information to address a particular situation.

Hence, our complex intervention consists of all the following simpler interventions: 1) Offering breastfeeding education and counseling to improve knowledge and expectations; 2) building of appropriate breastfeeding skills to improve self-efficacy and empower breastfeeding mothers; 3) providing professional lactation support; 4) establishing a mother-to mother tree of lay support. This intervention will develop new social network linkages and will use members of women’s own social networks to enhance their role in breastfeeding support.

To our knowledge, this is the first study of its kind to be conducted in Lebanon and the region as no previous studies investigated the effectiveness of a complex intervention composed of several simpler interventions previously shown to improve breastfeeding rates in low- or middle-income countries. Should this intervention prove to be effective, it can be used as a framework for a model intervention that may be replicated in any setting or community in Lebanon, whether rural or urban.

## Methods/Design

### Study design

A randomized controlled single-blind parallel-arm clinical trial to investigate whether a complex intervention targeting new mothers’ breastfeeding knowledge, skills and social support within a Social Network and Social Support theory framework will increase exclusive breastfeeding duration among women in Lebanon.

### Study population

Healthy pregnant women who are in their first or second trimester and who intend to breastfeed after delivery will be eligible to participate in this study.

Women with any of the following conditions will be excluded: pregnancy beyond the second trimester, chronic medical condition, abnormal fetal screen (ultrasound/blood/amniocentesis), not intending to breastfeed, not living in Lebanon for at least six months after delivery, twin gestation, and preterm birth (at <37 weeks gestation).

### Recruiting process

#### Inclusion of pregnant mothers

Eligible pregnant women will be recruited from two health care centers in Beirut, Lebanon: the Women’s Health Center of the American University of Beirut Medical Center (AUBMC), and the Obstetrics Clinics of Sahel General Hospital (SGH).

#### Randomization

Eligible pregnant women will be randomly allocated to one of two parallel groups (experimental and control, 1:1 ratio), using a computer-generated stratified block randomization that is done by one of the co-investigators (HT) who is not involved in subject recruitment. The size of blocks will vary from 4 to 8 and the stratification will be by study site. Allocation concealment will be done to ensure that group assignment of the patients are revealed only after assessing the inclusion/exclusion criteria are verified and consent obtained. This will be done to avoid any bias that might be introduced by the investigator because of the knowledge of the next allocation group. A set of sequentially numbered opaque sealed envelopes will be prepared with the allocation group, as per the randomization list, specified inside.

### Description of the intervention

#### Control group

Subjects in the control group will receive standard prenatal and postnatal care that is usually offered to mothers at both study sites. At AUBMC, standard prenatal care includes prenatal classes that cover issues related to labor, delivery and breastfeeding. Women who wish to attend a prenatal session will have to self-register. After delivery, mothers are usually instructed on breastfeeding by the nurses and their pediatricians. At SGH, there are no structured prenatal classes and any education or training on breastfeeding is often done by the nurses and pediatricians in the hospital, and later during well-baby checkups.

#### Intervention group

Women in the experimental group will, in addition to standard clinical care, receive a complex intervention starting in early pregnancy till 6 months post delivery. The intervention is composed of the following elements: a) prenatal breastfeeding education to raise knowledge and awareness, b) postpartum professional lactation support to improve maternal skills and self-efficacy, c) postpartum peer (lay) support to build social support, and enhance social capital within women’s social networks. These include skill building activities for the provision of effective breastfeeding support.

#### Details of the complex intervention

1. Prenatal breastfeeding education:

a. *Antenatal classes:* Upon enrolment and signing of the written informed consent, each subject in the intervention group will be invited to attend at least one antenatal session with as many members of her family as she wishes. Those sessions are meant to be a forum to discuss breastfeeding information and to address the family’s questions. They will be offered on a scheduled basis and open to the intervention group only in order to avoid contamination with the control group. Details of the objectives, content, format and description of the antenatal class appear in APPENDIX-A. Data on socio-demographic variables, baseline breastfeeding knowledge, behavior and attitude towards breastfeeding will be collected at the beginning of each session from each subject. We will use a “Breastfeeding Knowledge, Attitude, and Behavior” questionnaire (B-KAB) that will be developed and adapted from the following validated instruments: the Iowa Infant Feeding Attitude Scale (IIFAS) [25], the Infant Feeding Intention Scale (IFI) [26], the Breastfeeding Behavior Questionnaire (BBQ) [27,28], and the Infant Feeding Knowledge Test [29]. The B-KAB questionnaire will be developed through a discussion among all investigators for adjustments before generating the first version. After reaching a consensus, two experts in breastfeeding will be asked to review the questionnaires for content validity and cultural appropriateness. It will then be translated to formal Arabic (Grade 4 literacy level) by an expert translator. The Arabic questionnaires will be back translated to English by another independent translator. The two translations will be checked independently by two bilingual investigators for accuracy of translation. Following content validity, field testing will be conducted on 20 women to inquire about the questionnaire’s clarity and ease of comprehension. Any concerns, comments or suggestions will be noted, and necessary changes will be made before generating the final Arabic B-KAB questionnaires. Test-retest reliability will be established using a group of 20 students. Pearson correlation coefficient > 0.7 will be considered acceptable. Validation of the Arabic versions will be tested on the first 200 subjects enrolled in the study.

b. *Breastfeeding pamphlet and Video:* At the end of each antenatal class, each subject in the intervention group will get an educational pamphlet and a video that contains information on breastfeeding benefits, techniques and expectations, as well as information on formula hazards. Subjects will be instructed to share both resources with their family members, particularly husbands, mothers and mothers-in-law.

2. Professional lactation support:

In order to improve their breastfeeding skills and self-efficacy, mothers in the intervention group will be visited on a daily basis by a trained lactation expert (professional lactation support) when admitted to the hospital for delivery. Visits will be 15–30 minutes long and will entail hands-on training on breastfeeding positioning and latch as well as breast care and common breastfeeding concerns. To ensure breastfeeding continuity after hospital discharge, mothers will be visited in their homes on days 1, 3, 7 and 15, and then monthly until the 6th month postpartum, breastfeeding discontinuation, or until the mother requests that they stop, whichever occurs first. The lactation expert will be provided with a telephone to provide a 24-hour hotline service for additional breastfeeding support, and will answer questions relating to breastfeeding or refer to a physician when appropriate.

3. Social network and social support:

To enrich a breastfeeding mother’s social networks and strengthen her social support, we will establish mother-to-mother networks intended to provide breastfeeding mothers in the intervention group (BFMi) with lay/peer support. Support mothers (SM) will be recruited via three different methods:

a. Enhancing existing social network linkages: Each enrolled participant will be asked to identify 1 – 3 women in her community or family whom she believes can serve as a source of support to her efforts to breastfeed successfully. Each BFMi will be asked to contact potential SMs and request they contact study recruiters for possible enrollment. Recruiters will receive potential SM calls and set a time and place to conduct an interview with the research team. During the interview, the mother-to-mother support program will be explained. Candidates possessing the necessary skills and willing to be support mothers will then sign a written informed consent and thus will be enrolled. Inclusion criteria of support mothers are: history of successful breastfeeding of at least one child for 2 months, positive feelings about breastfeeding, able to attend two half-day training sessions to learn how to support new mothers and when to refer to professional resources, and can read and write Arabic (middle school level).

b. Developing new social network linkages: Flyers inviting women to become support mothers will be posted on bulletin boards of AUBMC pediatric and obstetric clinics as well as SGH obstetric and pediatric clinics after their permission. Eligible candidates enrolled in this manner will be matched with intervention subjects who were not able to identify possible SMs in their community and/or those whose nominated candidates were not considered suitable by the research team. The matching process will be based on age, availability and geographical proximity. Similar to above, clinic patients who are interested in becoming support mothers will be referred to the research team by their primary care physicians.

c. Snowball effect: Each enrolled SM will be asked to identify 1 – 3 women in her community or family whom she believes could serve as a good source of breastfeeding support to other mothers. She would then ask her/them to contact study recruiters for possible enrollment and consent. After receiving the potential SM’s call, the recruiter will set a time and place to conduct the enrollment interview. During the interview, the mother-to-mother support program will be explained; candidates possessing the necessary skills and willing to be support mothers will be enrolled.

Using these methods, we hope to enroll close to 74 SMs or approximately 1SM for every 2–3 BFMi. Breastfeeding support will occur in an informal manner based on a minimum number of scheduled calls/visits as follows:

1. Face-to-face contact during first antenatal class

2. Telephone call at beginning of the 6th month of gestation

3. Telephone call at beginning of the 9th month of gestation

4. Telephone call during the expected week of delivery

5. Hospital visit on the first day postpartum

6. Home visit at/telephone call 48 hours from discharge

7. Home visit/telephone call at 1 week postpartum

8. Home visit/telephone call at 2 weeks postpartum

9. Home visit/telephone call at 4 weeks postpartum

10. Monthly home visits/telephone call till 6 months postpartum

**Figure 1:**
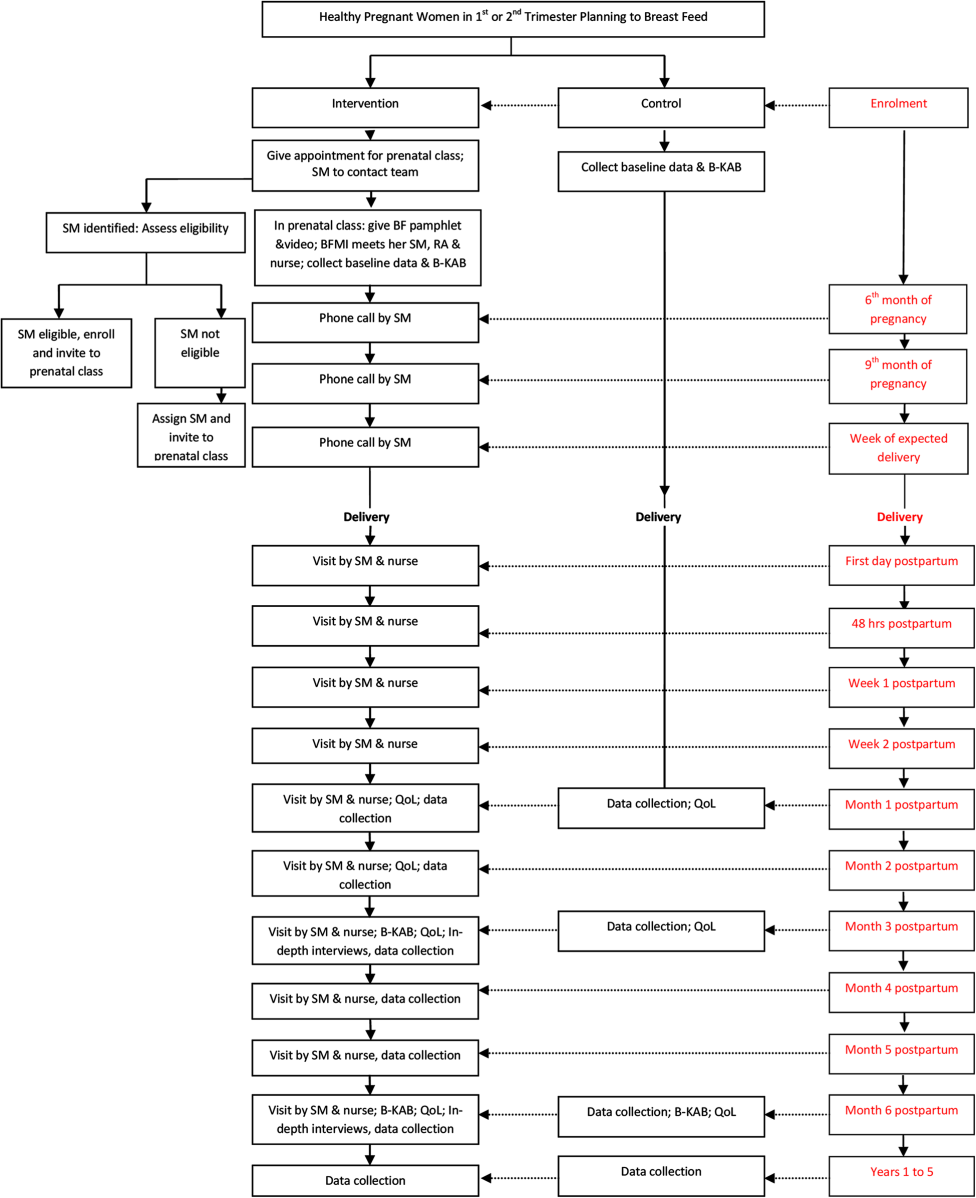
Flow diagram of participants through the trial.

Each BFMi will be given detailed instructions for contacting her SM when going to the delivery suite or immediately after delivery. Employed mothers who need to go back to their jobs after 4–6 weeks from delivery may be visited a week before the end of their maternity leave if they so wish. This schedule can be modified based on the needs of the BFMi. After each visit or call, support mothers will document details of the contact in the support mother activity record. Peer support will continue until the baby is 6 months of age or until the breastfeeding mother decides to stop, whichever comes first. Figure 1 details the flow of participants from recruitment till the last follow up contact for control and intervention subjects.

### Outcome measures

#### Main outcome measure

The primary outcome is the percent difference in 6-month breastfeeding exclusivity rates between the intervention and control groups.

### Secondary outcome measures

Differences between the two groups with respect to the following:

a) Breastfeeding exclusivity rates at 1 and 3 months.

b) Breastfeeding rates (exclusive or mixed feeding) at 1, 3 and 6 months.

c) Breastfeeding knowledge and attitudes of mothers at 6 months.

d) Success of a mother-to-mother support system measured as satisfaction rates of BFMis, SMs, and lactation experts; BFMi referral rates and adverse events during the 6 months.

e) Cost-benefit and cost-effectiveness analyses of the complex intervention.

f) Maternal quality of life at 1, 3 and 6 months using the validated Postpartum Quality of Life (QoL) questionnaire [30].

g) Success of mothers in exclusively breastfeeding new babies, measured as the percent difference between the intervention and control groups in 6-month breastfeeding exclusivity rates of subsequent babies, up to 5 years following the intervention.

### Recruitment

Trained recruiters will be present all day in the obstetric clinics at both sites, based on a specified schedule that is agreed upon with the obstetricians, depending on availability of clients. The study recruiter will identify eligible subjects visiting the clinics and approach them directly for participation in the study. She will explain the details and the procedure of the study, and check eligibility as per the inclusion and exclusion criteria. Subjects will be given the informed consent forms to read at leisure and will be encouraged to ask questions. They will also be given the recruiters telephone number should they wish to call later for consent or questions. Women will be interviewed in the privacy of a specified space dedicated for the study interviewers in each clinic.

### Data collection

#### Participant mothers

1. *Baseline:* Age, socio-economic status, education, employment status, mother’s religion, baseline breastfeeding knowledge and attitude, parity, previous breastfeeding, baseline social support, study site, residence.

2. *Month 1:* Baby’s gender, mode of delivery, pediatrician’s gender, first feed at hospital, rooming in, breastfeeding status, baby’s illness visits (number, diagnosis, money spent on medicine and doctor’s fees), baby’s hospitalizations (number, diagnosis, cost), days lost from work due to baby’s illness, days lost from work due to mother’s illness, cost of formula feeds/week, cost of water used to prepare formula/week, mother’s non-routine doctor visits due to breastfeeding, cost of infant food/week, QoL.

3. *Month 3:* Breastfeeding status, baby’s illness visits (number, diagnosis, money spent on medicine and doctor’s fees), baby’s hospitalizations (number, diagnosis, cost), days lost from work due to baby’s illness, days lost from work due to mother’s illness, cost of formula feeds/week, cost of water used to prepare formula/week, mother’s non-routine doctor visits due to breastfeeding, cost of infant food/week, QoL.

4. *Month 6:* Breastfeeding status, baby’s illness visits (number, diagnosis, money spent on medicine and doctor’s fees), baby’s hospitalizations (number, diagnosis, cost), days lost from work due to baby’s illness, days lost from work due to mother’s illness, cost of formula feeds/week, cost of water used to prepare formula/week, mother’s non-routine doctor visits due to breastfeeding, cost of infant food/week, QoL, satisfaction of mother with mother-to-mother support system, breastfeeding knowledge and attitude of mother, in-depth interviews with BFMis.

5. *Year 1:* Breastfeeding status, baby’s illness visits (number, diagnosis, money spent on medicine and doctor’s fees), baby’s hospitalizations (number, diagnosis, cost), days lost from work due to baby’s illness, days lost from work due to mother’s illness, cost of formula feeds/week, cost of water used to prepare formula/week, mother’s non-routine doctor visits due to breastfeeding, cost of infant food/week.

6. *Year 2:* Breastfeeding status, baby’s illness visits (number, diagnosis, money spent on medicine and doctor’s fees), baby’s hospitalizations (number, diagnosis, cost), days lost from work due to baby’s illness, days lost from work due to mother’s illness, cost of formula feeds/week, cost of water used to prepare formula/week, mother’s non-routine doctor visits due to breastfeeding, cost of infant food/week, breastfeeding status of new baby.

7. *Year 3:* Baby’s illness visits (number, diagnosis, money spent on medicine and doctor’s fees), baby’s hospitalizations (number, diagnosis, cost), days lost from work due to baby’s illness, breastfeeding status of new baby.

8. *Year 4:* Baby’s illness visits (number, diagnosis, money spent on medicine and doctor’s fees), baby’s hospitalizations (number, diagnosis, cost), days lost from work due to baby’s illness, breastfeeding status of new baby.

9. *Year 5:* Baby’s illness visits (number, diagnosis, money spent on medicine and doctor’s fees), baby’s hospitalizations (number, diagnosis, cost), days lost from work due to baby’s illness, breastfeeding status of new baby.

#### Support mothers

1. *Baseline:* Age, socio-economic status, education, employment status, religion, baseline breastfeeding knowledge and attitude, parity, previous breastfeeding.

2. *At end of support activity of each participant:* Activity log sheets, satisfaction with mother-to-mother support system.

3. *Year 1*: In-depth interviews with SMs.

#### Lactation experts

1. *Baseline:* age, socio-economic status, education, employment status, parity, previous breastfeeding.

2. *Month 6:* Satisfaction with the study experience.

### Data management and quality assurance

#### Training details

1. Professional lactation experts: Nurses recruited for professional support will have extensive training on breastfeeding in the WHO/UNICEF 20-hour course on breastfeeding as a minimum requirement. If not already certified as International lactation consultant, the nurse will be offered the exam to become certified. Once the trial is over, the lactation experts will help deliver structured training in breastfeeding for AUBMC and SGH nurses working in normal nursery, neonatal intensive care unit, delivery suite, maternity ward, and obstetric clinics. As such, we believe that investment in those experts will be cost-effective on the long run to help both sites achieve the “Baby-Friendly Hospital” status.

2. Recruiters: Two research assistants will be trained to approach and contact potential subjects at both sites. Training will entail familiarizing them with all necessary documentation, including enrollment and consent forms in a structured 2-hour workshop. They will be trained to consent mothers in accordance with the ethical principles of informed consent, and call them using a prepaid phone card. Instructions and demonstrations on how to use these cards will also be given during the same workshop.

3. Support mothers: Training of SMs will take place at AUBMC at an agreed upon time and place. One of 3 pediatricians -part of the research team- will conduct seven training workshops. Each workshop consists of two 2-hour sessions and will include a maximum of 10 SM participants. The first of the 2 sessions will be mainly theoretical. It will entail a brief overview of the mother-to-mother support program and training on the LOVE (listen observe validate empower/educate) method of support. In the second part of the first session, *breastfeeding basics* will be discussed, including advantages of breast milk and risks of formula; common culture-specific misconceptions will be addressed and proper technique of breastfeeding will be demonstrated using visual aid material. Breastfeeding trouble-shooting, and criteria for referral to appropriate medical services will also be emphasized. These criteria aim to identify mothers with medical conditions such as mastitis or breast abscess and postpartum depression or psychosis as well as infant medical conditions, such as hyperbilirubinemia and/or dehydration. During the second session, which will be largely practical, support mothers will be asked to review what they have learned through interactive discussions; they will engage in role playing of “*what if*” scenarios as necessary, including failure to breastfeed scenarios. For those specific cases, SM’s empathy and positive attitude will be emphasized. They will subsequently be handed a small manual containing a list of local breastfeeding resources as well as their telephone log sheets and activity records.

#### Process evaluation

During the support period, an ongoing evaluation process will take place in order to identify potential problems and implement changes accordingly. The process evaluation will ensure that the intervention is delivered and implemented as planned by evaluating the dose delivered, dose received, and reached.

The study research assistant will contact BFMis and SMs bi-weekly to inquire about the support process, including address potential complaints. The inquiry findings will be shared with the principal investigator within one business day. Process amendments and/or improvements will take place within the same week if necessary. In case of a perceived need for referral by SMs, physician members of the research team will be notified within the same day and will contact the BFMi-SM pair in question to evaluate and confirm the medical urgency and refer-or not- accordingly. At the end of the support period, all records are returned to the research team. When the latter receives the records, a post-support survey is sent to the breastfeeding mother to fill and return for data entry and analysis. Separate surveys will be sent to each support mother and to the professional nurses to assess their satisfaction. All BFMis will be contacted by the research team on yearly basis for 5 years after the trial ends to ask about their success in breastfeeding future babies. In addition, all mothers in the control group will be contacted by the research team at 1 month, 3 months and 6 months after delivery for data collection and outcome assessment similar to the intervention group. They will also be contacted on yearly basis for 5 years after the trial ends to ask about their success in breastfeeding future babies.

### Sample size

The most recently reported 6-month exclusive breastfeeding rate in Lebanon is 2% [23], which is the expected rate in the control group. The complex intervention is hypothesized to increase the 6-month exclusivity rate in the intervention group to 12%. To detect this 10% difference between the two groups with 90% power and 5% type I error, 155 mothers are needed per group. Allowing for a loss-to-follow up rate of 30%, the needed sample size becomes 443 women. The number of SMs to be recruited is 74, assuming that each support mother will pair with 3 breastfeeding mothers from the intervention group.

### Statistical methods

We will compare continuous variables using Student’s *t* test and categorical variables using Chi square test. The relationship between breastfeeding exclusivity as outcome and the independent variables (maternal age, study group, socio-economic status, maternal education, study site, pediatrician’s gender, type of delivery, baby’s gender, parity, previous breastfeeding, maternal employment, mother’s religion, baseline breastfeeding knowledge and attitude, baseline social support) will be explored in bivariate and multivariate analysis. Regression models will be built to adjust for possible confounding in the relationship between the dependent and the independent variables stated above. All analysis will be done on intention to treat basis. The Statistical Package for Social Sciences (SPSS) will be used for data management and analyses. A p-value of <0.05 will indicate statistical significance.

### Cost analysis

The economic efficiency of breastfeeding promotion will be investigated through the assessment of the costs incurred during 1 year by families feeding their infants artificial milk versus breastfeeding (whether exclusive or mixed). The analysis will take into consideration the cost of planning and implementing the Complex Intervention for Breastfeeding Promotion initiative and the various costs associated with healthcare provision, nutrition and lost productivity during one year for families in both study groups. The cost analysis will test whether the economic burden of exclusive breastfeeding is less than partial breastfeeding, and less than formula feeding at the family level, as well as at the national level. We will explore the association between the infant feeding status (exclusive breastfeeding, partial breastfeeding, formula feeding), and study group (intervention/control) as independent variables, and the following dependent variables: non routine doctor visits, hospitalization, work absences due to infant sickness, work absences due to mother sickness, cost of formula milk, cost of water used for formula preparation, mother’s non-routine doctor visits related to breastfeeding, lost productivity of parents due to breastfeeding, and cost of other infant foods.

### Ethical approval

The study is approved by the Internal Review Boards of the American University of Beirut and Al-Sahel General Hospital. All participant pregnant women and support mothers will be requested to give their written informed consent prior to any study procedure. To secure confidentiality, all identifying information of participants, including name, medical and contact information as well as all collected data will be kept in a password protected computer that is kept secure in a locked cabinet by the principal investigator. Data access will be limited to the principal investigators and project coordinator working directly on the study.

To ensure appropriate medical and /or psychiatric care is provided to those mothers who need it, an ongoing evaluation process will take place with referral to appropriate services after physician notification and evaluation.

SM training will emphasize empathy and encouragement in order to ensure that breastfeeding mothers do not feel pressured to continue breastfeeding if they do not wish to do so. BFMis who fail to continue exclusive breastfeeding will continue to receive positive messages and get support if they so wish. This will continue until six months postpartum or until the BFMi decides to stop, whichever comes first. Mothers in the control group will receive the breastfeeding pamphlet and video upon exiting the study.

### Associated studies

1. Sub-study 1: The mother-to-mother breastfeeding support pilot study.

2. Sub-study 2: Development and validation of the Arabic breastfeeding knowledge, attitude and behavior (B-KAB) questionnaire.

3. Sub-study 3: Assessment of the impact of the complex breastfeeding intervention on knowledge and attitudes of breastfeeding mothers and their families.

4. Sub-study 4: A qualitative study on the experiences of breastfeeding mothers and support mothers participating in the complex breastfeeding promotion and support intervention trial.

5. Sub-study 5: Professional nurses experiences and satisfaction with the breastfeeding promotion and support intervention.

6. Sub-study 6: Impact of the complex breastfeeding intervention on the quality of life of participating mothers.

7. Sub-study 7: Cost-benefit and cost-effectiveness analyses of the complex breastfeeding intervention.

8. Sub-study 8: Development and validation of the Arabic Postpartum Quality of Life Questionnaire.

## Discussion

This study aims at investigating whether a complex intervention targeting new mothers’ breastfeeding knowledge, skills and social support within a Social Network and Social Support theory framework will increase exclusive breastfeeding rate and duration among women in Lebanon. We anticipate that the proposed multi-dimensional intervention will increase the low breastfeeding rates in Lebanon as it addresses previously identified barriers to successful breastfeeding in this country, namely inadequate maternal skills and self-efficacy, cultural misconceptions about breastfeeding, and lack of adequate social support. We believe that this unique multi-dimensional approach is a main strength of our study. Other strengths include the planned assessment of multiple outcomes over a five year period including quality of life, economic efficiency and maternal success in breastfeeding future babies, which have not been previously assessed in a clinical trial context.

The study may suffer from some limitations. The first limitation is the sampling frame which is the population of mothers who intend to breastfeed rather than all the population of expectant mothers. This frame was chosen because inclusion of women who do not intend to breastfeed will necessitate a much larger sample size, with further inflation of the budget thus making the study unfeasible. This selection bias however may exaggerate the difference in the 6-month exclusivity rates between the control and intervention groups. Another limitation is the single-blind design which is due to the difficulty in concealing the intervention from the research team members who will be directly involved in delivering the different aspects of the breastfeeding support package. However, this bias is unlikely to affect the main outcome of the study, the 6-month exclusivity rate, because of its objective nature.

The proposed complex intervention, if proven to be effective, will have a significant impact on infant morbidity and mortality, as well as maternal health and quality of life. Moreover, the study is expected to generate useful data that will encourage replication of the model in Lebanon as well as in similar developing countries. This study will highlight the compounding effect of using multiple methods to support breastfeeding mothers by utilizing the social network to promote exclusive breastfeeding.

## Appendix-A

### Antenatal breastfeeding education class

**Purpose:** To promote breastfeeding among expectant mothers and improve their breastfeeding-related knowledge and skills.

**Description:** A one-hour session of educational activities that aim at promoting breastfeeding among expectant mothers and their families.

### Objectives

At the end of the session the expectant mother will be able to:

1. Identify the importance of breastfeeding to mother and baby

2. Know the mechanism of milk production

3. Practice adequate breastfeeding technique, including recognize different breastfeeding positions and proper latch

4. Understand the concept of rooming in and its benefits

5. Adapt the concept of breastfeeding maintenance after return to work

6. Identify concerns related to breastfeeding and how to overcome them

### Course content

1. Why is breastfeeding important

2. Benefits of breastfeeding to mother and baby

3. Common breastfeeding myths

4. How does breastfeeding work (anatomy and physiology of milk production)

5. Baby’s readiness to eat

6. Techniques of a good latch

7. Different positions during breastfeeding

8. Breast care

9. Preventing soreness and engorgement

10. Rooming-in to promote breastfeeding

11. Working mothers and breastfeeding

12. Family support during breastfeeding

13. Common concerns related to breastfeeding

### Course material

1. Power point presentation

2. Handouts

3. Demonstration

### Teaching aids

1. Doll

2. Breast Model

3. Others if needed

### Teaching formats

1. Interactive discussion

2. Case study

3. Pre- and Post- Assessment checklist

4. Role play

## Abbreviations

SSPS: Statistical package for social sciences; MENA: Middle East and North Africa; AUBMC: American University of Beirut Medical Center; SGH: Sahel General Hospital; KAB: Knowledge-Attitude-Behavior; IIFAS: Iowa infant feeding attitude scale; IFI: infant feeding intention scale; BBQ: Breastfeeding behavior questionnaire; SM: Support mother; BFMi: Breastfeeding mother in intervention group; QoL: Quality of life; WHO: World Health Organization; UNICEF: United Nations International Children’s Emergency Fund; LOVE: Listen observe validate empower/educate.

## Competing interests

The authors declare that they have no competing interests.

## Authors’ contribution

MN was responsible for the conception and design of the study, questionnaires, and data collection instruments, wrote the first and final versions of the protocol, and applied and received grant for the study. HH was responsible for the conception and design of the study and questionnaires, and participated in the writing of the initial and final protocol versions. HT participated in study design, writing of statistical section and translation of questionnaires. TK participated in study conception and design, and writing of the Social Network and Social Support Theory framework. LC participated in designing and writing the intervention related to breastfeeding knowledge. NY participated in study design and writing of the cost efficiency and the breastfeeding knowledge sections. DS and SM participated in study design and writing of the breastfeeding skills section. All investigators contributed to all drafts of the study and critical review of the final protocol version.

## Pre-publication history

The pre-publication history for this paper can be accessed here:

http://www.biomedcentral.com/1471-2458/14/36/prepub
